# Modulation of Multiple Signaling Pathways of the Plant-Derived Natural Products in Cancer

**DOI:** 10.3389/fonc.2019.01153

**Published:** 2019-11-08

**Authors:** Li-rui Sun, Wei Zhou, Hong-mei Zhang, Qiu-shi Guo, Wei Yang, Bing-jin Li, Zhi-hui Sun, Shuo-hui Gao, Ran-ji Cui

**Affiliations:** ^1^Department of Pharmacy, The First Hospital of Jilin University, Changchun, China; ^2^Jilin Provincial Key Laboratory on Molecular and Chemical Genetic, The Second Hospital of Jilin University, Changchun, China; ^3^Department of Gastrointestinal Colorectal Surgery, China-Japan Union Hospital of Jilin University, Changchun, China

**Keywords:** natural active compounds, signaling pathway, cancer, polyphenol, alkaloid, saponin, polysaccharide

## Abstract

Natural compounds are highly effective anticancer chemotherapeutic agents, and the targets of plant-derived anticancer agents have been widely reported. In this review, we focus on the main signaling pathways of apoptosis, proliferation, invasion, and metastasis that are regulated by polyphenols, alkaloids, saponins, and polysaccharides. Alkaloids primarily affect apoptosis-related pathways, while polysaccharides primarily target pathways related to proliferation, invasion, and metastasis. Other compounds, such as flavonoids and saponins, affect all of these aspects. The association between compound structures and signaling pathways may play a critical role in drug discovery.

## Introduction

In 2018, an estimated 9.6 million deaths were caused by cancer, and cancer is anticipated to be the leading cause of death worldwide in the twenty-first century (1). Therefore, cancer prevention remains an innovative area of anticancer research, in addition to cancer therapy. The mechanisms of aberrant signal transduction pathways in cancer and the impacts of these pathways on tumorigenesis, apoptosis, and metastasis have been increasingly revealed due to intensified study (2). Searching for targeted molecules that can regulate signal transduction has recently emerged as a globally popular research area in biomedicine.

Herbal medicines, such as Chinese medicines, are naturally exceptional at ameliorating many human diseases. Increasing numbers of new drugs with pharmacological activity have been discovered due to the modernization of herbal medicine. The anticancer agents vincristine, taxol, and vinblastine have been used for their anticancer effects in many countries (3). Moreover, other promising anticancer agents are available, including arteannuin (4), quercetin (5), and tetrandrine (6). Alkaloids and polyphenols are significantly dominant among cancer therapeutics (7, 8). Recently, the targets and mechanisms of plant-derived anticancer agents have been widely reported (9). In this review, we will focus on advances in knowledge about the signaling pathways affected by plant-derived natural products.

## Polyphenols

Polyphenols are particularly ubiquitous in vegetables, fruits, and other foods. Thousands of polyphenols have been identified (10), and these compounds have broad-spectrum pharmacological activities including anticancer effects. Polyphenols can be classified by their chemical structures into several classes such as flavonoids, xanthones, stilbenes, lignans, and curcuminoids (Table 1) (11–14). Many natural polyphenols have cytostatic and apoptotic properties because of their antioxidant characteristics (11). The anticancer effects of polyphenols depend not only on their chemical structure and concentration but also on the type of cancer. Lignans considered to be phytoestrogens are bioactive compounds exhibiting various anticancer properties, such as apoptosis induction and tumor growth reduction (15). Xanthones, such as α-mangostin, mediate cytotoxicity mainly via cell cycle arrest and reactive oxygen species (ROS)-induced apoptosis (16). The anticancer effects and molecular mechanisms of polyphenols are reported to be associated with their chemical constitution which is necessary for its anticancer activities, such as the C-3 prenylation of benzoxanthone-type prenylated flavonoids, C-1 hydroxy group and isoprenyl group at C-8 of prenylated xanthones, the C-2 carbonyl group, C-4 prenyl group and pyran ring connected at the C-2 and C-3 of caged xanthones (9). Anticarcinogenic activities of polyphenols include suppressing the proliferation, differentiation, metastasis, and angiogenesis of various kinds of cancer cells through inhibiting several kinases involved in signal transduction (17–20). Polyphenols can bind and cross cell membranes easily and trigger various pathways involving microRNAs (miRNAs), caspases, B cell lymphoma 2 (Bcl-2) family proteins, nuclear factor (NF)-κB, epidermal growth factor (EGF)/epidermal growth factor receptor (EGFR), phosphatidylinositol-3-kinase (PI3K)/Akt, mitogen-activated protein kinase (MAPK) (Table 2).

**Table 1:** Classifications of polyphenols.

**carbon units**	**Classifications**	**Components**
C6-C3-C6	Flavonoids	Chrysin, silibinin
C6-C1-C6	Xanthones	α-mangostin
C6-C2-C6	Stilbenes	Resveratrol
C6-C3-C3-C6	Lignans	Podophyllotoxin
C6-C3-C1-C3-C6	Curcuminoids	Curcumin

**Table 2:** Polyphenols and their anticancer mechanisms.

**Mechanism**	**Components**	**Plant origin**	**Cell line**	**References**
PKC/MAPK signalway ↓	Trichosanthin	*Trichosanthes kirilowii* Maxim	K562 HeLa	(21)
β3 integrin/FAK signalway ↓	Tuteolin	dragonhead	B16F10	(22)
fatty acid synthase (FAS) ↓	Epigallocatechin-3-gallate	green tea	LNCaP	(23)
STAT3 ↓	Tectochrysin	*A. oxyphylla* Miquel	NCI-H460 A549	(24, 25)
MAPK/ERK signalway	silibinin Fisetin Genistein licochalcone A Apigenin pterostilbene	*Silybum marianum* fruits and vegetables soy licorice root fruits and vegetables grapes, blueberries	A549 PC12 PC3 BGC-823 Leukemia cells Breast cancer	(26) (27) (28) (29) (30) (31)
Akt signalway ↓	Chrysin	celery	U87-MG U-251	(21)
EGFR tyrosine kinase	Luteolin Quercetin	Dragonhead Quercus	A431	(22)
EGFR/MEK/ERK signalway ↓	Arctigenin	*Arctium lappa*	Tissues from gallbladder cancer patients	(32)
Akt/mTOR signalway	Fisetin	Fruits and vegetables	U266	(23)
Bcl-2 ↓	Fisetin	Fruits and vegetables	U266	(33)
	Ampelopsin	*Ampelopsis grossedentata*	LNCaP PC3 Animal model	(34)
X-linked inhibitor of apoptosis protein (XIAP)↓	chrysin	celery	U937	(35)
PI3K/Akt signalway	Licochalcone A Pterostilbene Arctigenin	licorice root grapes, blueberries *Arctium lappa*	BGC Breast cancer LNCaP	(35) (31) (36)
DNA topoisomerase II	podophyllotoxin	rhizomes of *Podophyllum* species	Ehrlich ascites tumor cells	(37)
V-ATPase↓mTORC1/HIF-1α-/VEGF signalway↓	Diphyllin	*Cleistanthus collinus*	TE-1 ECA-109	(38)
NAPDH oxidase-5/ROS↑	Resveratrol	Red wine and grapes	NSCLC	(39)
ASK1/p38 signalway	α-mangostin	*Garcinia* *mangostana Linn*	SiHa and HeLa	(40)
miR-21, miR-15a, miR-141, miR-155, miR-125b and miR-182↓miR-200c↑	silibinin	*Silybum marianum*	MDA-MB-231 MCF-7 T47D	(41–43)

### MicroRNAs

MicroRNAs (miRNAs) are small non-coding RNAs (NC-RNAs) and regulate gene expression via binding to 3′ untranslated regions (UTRs) of target mRNA (44). Approximate 1,500 miRNA have been identified in the human (45). Oncogenic miRNAs have been identified in many kinds of cancers such as miR-7-1, miR-21, miR-92, miR-122, miR-125b, miR-155, miR-330 (46). It is indicated miRNAs are critical in cancer cell proliferations, differentiation, apoptosis, and invasion through the regulation of oncogenic gene expression (47, 48). It is predicted a miRNA can recognize an average of 100–200 different mRNA targets (49, 50). For example, miR-155 modulates the expression of NF-κB and MAFK via regulation of BACH1 (BTB and CNC homology 1, basic leucine zipper transcription factor 1) and LDOC1 (leucine zipper, downregulated in cancer 1) which is critical to malignant transformation in leukemia, breast and lung cells (51–53). It is emphasized that miRNAs are novel therapeutic targets of polyphenols such as curcumin, resveratrol, genistein, EGCG and silibinin (45, 54–56).

Curcumin [(1,7-bis(4-hydroxy-3-methoxyphenyl)-1,6-heptane-3,5-dione] is a curcuminoid extracted from the rhizome of *Curcuma longa* Linn (57). It is demonstrated that 5–40 μM of curcumin has effects on a variety of miRNAs in different cancer cell lines such as miR-192-5b (58), miRNA-98 (59), miR-21 (60–62), miR-15a (63, 64), miR-101 (65, 66) in lung cancer, colorectal cancer, leukemia, colon cancer, and breast cancer to inhibit cell viability and metastasis, induce apoptosis.

According to quantitative reverse transcription-polymerase chain reaction (qRT-PCR) analysis, resveratrol (3,4′,5-trihydroxy-trans-stilbene) with dosage of 10–150 μM induces apoptosis and depresses cell proliferation, invasion via inhibition of NF-κB activity, Akt/Bcl-2 pathway, EZH2 pathway, STAT3 and COX-2 activity through upregulation of miR-34a (67), miR-326 (68), miR-200c (69), miR-137 (70), and miR-328 (71), and downregulation of miR-19 (72), miR-21 (73), miR-196b (74), miR-1290 (74), and miR-221 (75, 76).

**Figure 1:**
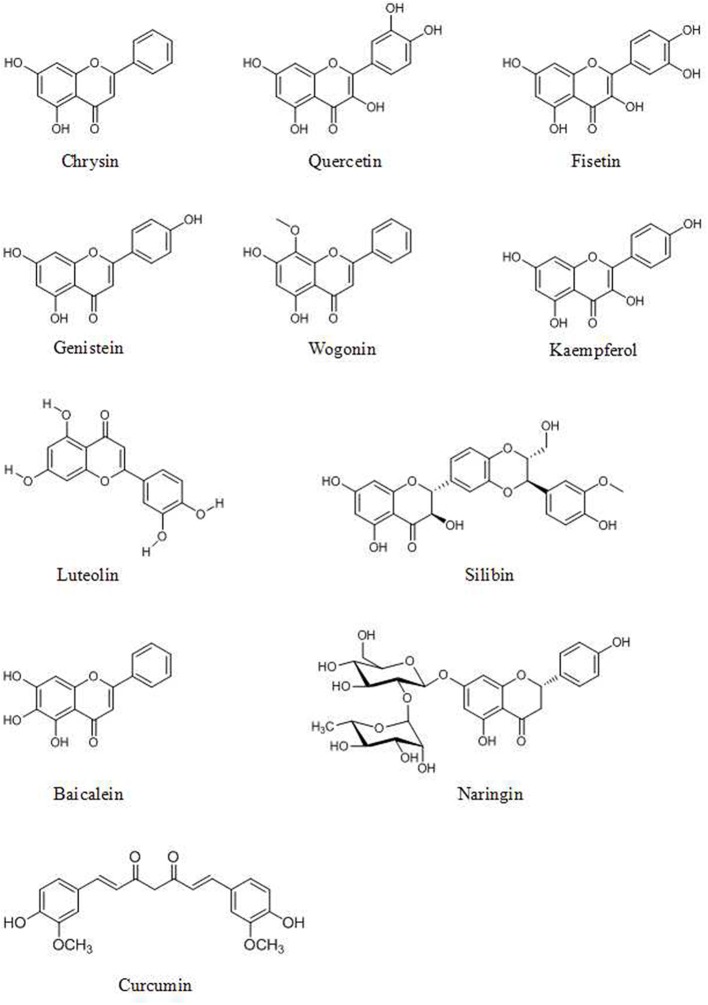
Chemical structures of some flavonoids.

Genistein (4′,5,7-trihydroxyisoflavone, Figure 1), found in soy products, has effects on miRNAs in various cancer cells (77). Breast cancer cell growth is inhibited by the induction of miR-23b and inhibition of miR-155 by 25–175 μM of genistein treatment (78, 79). Genistein inhibits the expression of miR-27a (80) and miR-223 (81) and induces the expression of let-7d (82) and miR-34a (83) which play an important role in pancreatic cancer cell growth and invasion. Genistein also exerts its anticancer activity via upregulation of miR-200c (84) and downregulation of miR-151 in prostate cancer (85).

The green tea extracts (–)-epigallocatechin (EGC) and (–)-epigallocatechin-3-gallate (EGCG) also targets oncogenic miRNAs including upregulation of miR-16, let-7a, and miR-221 and downregulation of miR-18a, miR34b, miR-193, miR-222, and miR-342 in human hepatocellular carcinoma cells (86). Expression of miR-548m and miR-720 are down-regulated in human breast cancer MCF-7 cells (87). miR-210 is up-regulated by EGCG in lung cancer cells which is associated with HIF-1α (hypoxia-inducible factor 1-alpha) (88). EGCG (40–60 μg/ml) suppresses cell growth of cervical carcinoma by regulation of miRNAs including up-regulation of miR-29, miR-29a, miR-203 and miR-210, and down-regulation of miR-125b, miR-203, miR-125b (89).

### NF-κB Pathways

NF-κB can regulate the transcription of genes associated with the inflammatory response, cell death, and proliferation (90, 91). NF-κB pathways participating in the development of various cancers can be disrupted by polyphenols. The PI3K/Akt signaling pathway and MAPK signaling pathways are related to the activation of NF-κB in numerous tumor cell lines (92).

**Figure 2:**
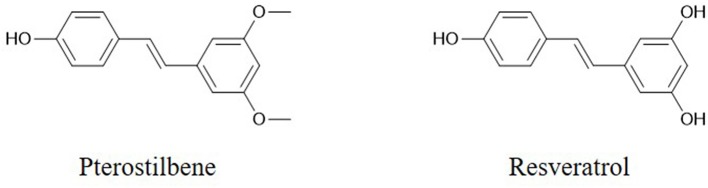
Chemical structures of some stilbenes.

The flavonoid component chrysin (5,7-dihydroxyflavone, Figure 1) has been shown to suppress the growth of colon cancer cells via direct inhibition of NF-κB expression and activity, according to computational docking experiments (24). In addition, 30 μM chrysin activates NF-κB/p65 by inducing p38 MAPK signaling pathways in HeLa cells (33). Quercetin (Figure 1) has a potential role in inhibiting processes in human oral cancer cells through the NF-κB pathway (93). The results of Western blot and flow cytometric assays indicate that the flavonoid fisetin (3,3′,4′,7-tetrahydroxyflavone, Figure 1) effectively suppresses the apoptosis, metastasis, angiogenesis and invasion of cancer cells via ERK1/2-, Akt/NF-κB/mTOR- and p38 MAPK-dependent NF-κB signaling pathways (94, 95). Furthermore, fisetin is not cytotoxic to normal cells (94). Genistein has a potential role in inhibiting cell division and apoptosis via Akt and NF-κB (28). Wogonin (Figure 1), extracted from *Scutellaria baicalensis Georgi*, can decrease the phosphorylation levels of IκB and p65. Modulation of the NF-κB/Bcl-2 signaling pathway has been shown by Western blot analysis to play a critical role in both of the invasion and proliferation of hepatocellular carcinoma (HCC) in a dose-dependent manner (96). Wogonin is shown to decrease the protein and mRNA levels of cyclooxygenase (COX)-2 in skin fibroblast NIH/3T3 cells and in animal experiments (97). The stilbene pterostilbene (*trans*-3,5-dimethoxy-4-hydroxystilbene, Figure 2), the dimethylated analog of resveratrol, is a highly bioactive natural polyphenolic compound that is mainly found in grapes, blueberries, tomatoes, and other berries (98). According to the results of COX-2 activity assays and enzymatic immunoassays, both resveratrol (Figure 2) and pterostilbene cause COX-2 inactivation via the NF-κB signaling pathway (31, 99).

### Matrix Metalloproteinase (MMP)-2 and MMP-9

The MMPs are a group of metal-dependent proteolytic enzymes that are involved in matrix remodeling and facilitate the migration of cancer cells through degradation of the extracellular matrix (100). MMP-2 and MMP-9 can degrade type IV collagen in the basement membrane and facilitate tumor cell metastasis (101).

Various polyphenols affect MMPs. Some, such as 5 μM resveratrol (102) and 75–100 μM kaempferol, inhibit the activity of MMPs (Figure 1) (103). Others decrease the expression of MMPs. The flavone luteolin (Figure 1) inhibits colon cancer metastasis by reducing the expression of MMP-2 and MMP-9 (104). The flavonolignan silibinin (C_25_H_22_O_10_, Figure 1), an active compound of *Silybum marianum* (L.) Gaertn, decreases the expression of MMP-2, MMP-3 and MMP-9 and increases the expression of TIMP-2 in prostate tumor tissue in transgenic adenocarcinoma of the mouse prostate (TRAMP) model mice and *in vitro* in various cancer cells (26, 104, 105). MMP-2 expression is downregulated in human prostate cancer cells by genistein treatment (28). In addition, treatment with 5 μM quercetin and chrysin decreases the expression of MMP-9 in A549 cells (106). Still other polyphenols affect both the activity and expression of MMPs. For example, naringin (4',5,7-trihydroxyflavanone 7-rhamnoglucoside, Figure 1) can inhibit the adhesion and invasion of human glioblastoma U87 cells and U251 cells via dose-dependent reductions in both the activity and expression of MMP-2 and MMP-9, according to zymograohy and Western blotting results, this effect is associated with the p38 MAPK signaling pathway (107, 108). EGCG (20 μM) reduce the activity of MMP-2 and MMP-9 in prostate cancer cells (109) and decrease the expression of MMP-9 in bladder cancer cells (110).

### Caspases

**Figure 3:**
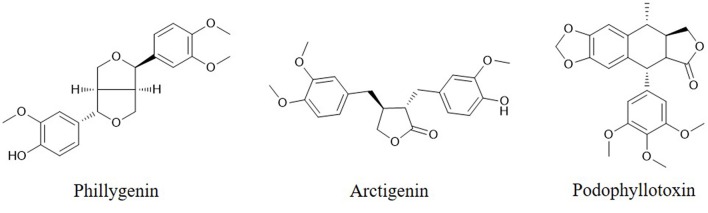
Chemical structures of some lignans.

**Figure 4:**
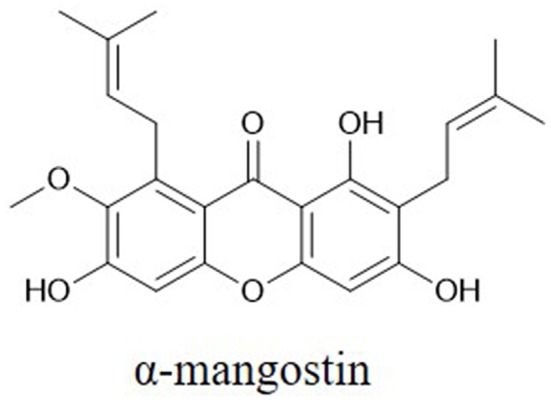
Chemical structures of some xanthones.

Caspases, which are activated by other caspases, are cysteinyl aspartate-specific proteases and are divided into two groups. One group comprises initiators (caspase-8, -9, and -10); the others, executioners (caspase-3, -6, and -7). Caspase -3 is considered the major downstream target of caspase-4, -8, and -9. Overexpression of caspases is a common alteration in cancer cells that can be exploited therapeutically. Activation of caspase-3 by fisetin treatment associated with induction of the proapoptotic proteins Bad, Bax, Bim, and inhibition of the antiapoptotic proteins Bcl-2 and Mcl-1(L) (35). Genistein has also been shown to increase the expression of caspase-3,-9 and Bax *in vitro* (28). Chrysin-induced apoptosis was associated with induction of caspase-3 and-8 and downregulation of phospholipase C-gamma-1 (PLC-gamma1) and XIAP. This finding suggests that the mechanism of apoptosis induced by chrysin is associated with Akt dephosphorylation in the PI3K signaling pathway (33). EGCG can induce apoptosis and reduce cancer cell proliferation by decreasing the mitochondrial membrane potential (ΔΨm) and stimulating caspase-3, -9 and c-Jun N-terminal kinase 1 (JNK1) expression in human glioblastoma T98G and U87MG cells but does not induce apoptosis in human normal astrocytes (111). The flavonoid baicalein (Figure 1), found in *Scutellaria baicalensis Georgi*, participates in apoptosis by increasing the expression of caspase-3 and -8 (112). The lignan phillygenin (Figure 3) induces apoptosis by increasing the mitochondrial membrane potential due to increased ROS levels in human esophageal cancer SH-1-V1 cells. Concurrent upregulation of Bax and cleaved caspase-3 and -9, along with dose-dependent downregulation of Bcl-2, was found by propidium iodide staining and Western blotting (15). The anticancer effects of arctigenin (Figure 3), the active component of *Arctium lappa*, are mainly directed toward cancer cell growth inhibition and apoptosis through the peroxisome proliferation-activated receptor α (PPARα)/gankyrin, Bax and caspase pathways (36). The xanthone α-mangostin (Figure 4) increases the activity of caspase-3 and causes late apoptosis in ovarian adenocarcinoma SKOV3 cells after 12 h and 72 h of treatment, respectively (113).

## Alkaloids

Alkaloids are the secondary biologically active components found in many plants. Alkaloids have various biological activities that render them important sources for drug discovery. The presence of nitrogen in their molecular architecture is critical to the biological activity of this class of compounds. Many studies have shown that alkaloids inhibit the growth of human breast, liver, colon, prostate, and liver cancer cells (114).

### Bcl-2 Protein Family

Bcl-2 proteins are divided into two groups. Bcl-2 and Bcl-xL are antiapoptotic proteins, while Bax and Bad are multidomain proapoptotic proteins. The balance of antiapoptotic proteins to proapoptotic proteins, for example, the ratio of Bax to Bcl-2 is crucial to the regulation of apoptotic pathways (115). The balance between Bcl-2 family proteins is a potential target of alkaloids for inducing cell death (116).

**Figure 5:**
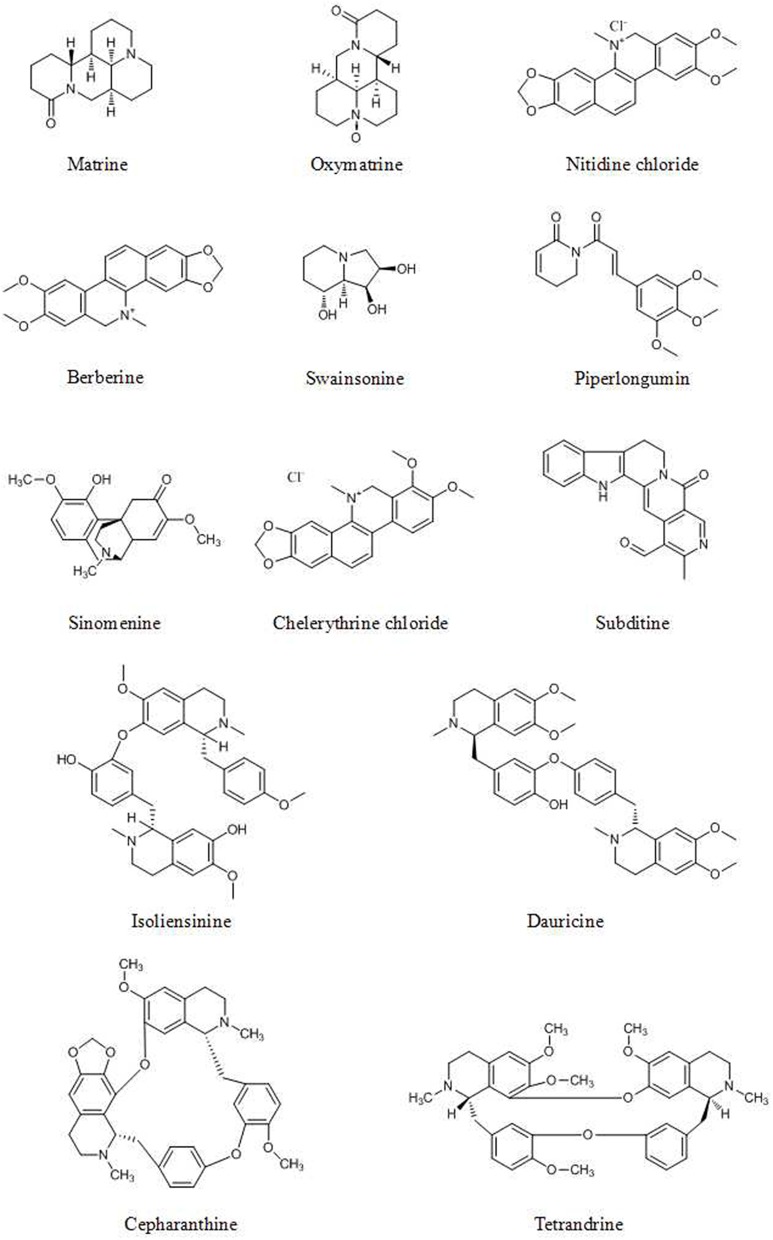
Chemical structures of some alkaloids.

Oxymatrine (Figure 5), derived from *Sophora flavescens* Aiton, significantly increases p53 and Bax expression and decreases Bcl-2 expression dose-dependently, as evidenced by A Western blot assay, in osteosarcoma cancer cells via dephosphorylation of PI3K and Akt in the PI3K/Akt signaling pathway (117).

Treatment with crude alkaloid extractof *Rhazya stricta* (CAERS) induced apoptosis and suppressed the proliferation of HCT116 cells. Downregulation of Bcl-2, survivin, Bcl-X and XIAP expression and upregulation of Bad and Noxa expression were examined by qRT-PCR and Western blot analyses and coincided with the increase in the Bax/Bcl-2 ratio (118).

Various alkaloids induce apoptosis via an increase in the Bax/Bcl-2 ratio. Cancer cells treated with nitidine chloride (NC, Figure 5), matrine (Figure 5), berberine (Figure 5), and subditine (Figure 5) showed upregulation of Bax expression and downregulation of Bcl-2 expression (119–123).

### PI3K/Akt/mTOR Signaling Pathway

Autophagy is a critical process for maintaining intracellular homeostasis. Generally, autophagy may play a critical role in cancer prevention (124). The PI3K/Akt/mTOR pathway is critical for autophagy induction and is a latent target in cancer therapeutics and control (101).

Piperlongumine (Figure 5) (125), swainsonine (Figure 5) (126), and sinomenine (Figure 5) (127) induce apoptosis and inhibit cancer cell growth through the PI3K/Akt/mTOR pathway, with decreased levels of p-Akt and p-mTOR, as evidenced by the results of Western blot analysis and immunofluorescence. Isoliensinine (Figure 5), matrine, dauricine (Figure 5), and cepharanthine (Figure 5) induce autophagy through the AMPK-TSC2-mTOR signaling pathway, with suppression of mTOR activity (128–130).

### ERK Signaling Pathway

The MAPK/ERK pathway participates in multiple processes in cancer including growth, invasion, metastasis, angiogenesis, and inhibition of apoptosis (131, 132). Because of these multiaspect effects, the MAPK/ERK pathway plays a critical role in the promotion of cancer cell growth and the inhibition of apoptosis (133, 134).

β-carboline alkaloids extracted from the seeds of Peganum harmala inhibit the proliferation and induce the apoptosis of SGC-7901 cells, possibly because β-carboline alkaloids can disrupt the balance between PTEN and ERK, inhibit the MAPK/ERK signaling pathway and induce apoptosis in cancer cells (135). Berberine can suppress the senescence of human glioblastoma cells by inhibiting the EGFR/Raf/MEK/ERK pathway (136). Sinomenine, extracted from *Sinomenium acutum*, is reported to inhibit various types of cancer cells. Sinomenine hydrochloride (SH) increases the phosphorylation of ERK1/2, p38 and JNK but does not affect the total levels of the abovementioned cytokines (137). The benzo phenanthridine alkaloid chelerythrine chloride (CC, Figure 5) (5 and 10 μM) significantly enhances ERK1/2 phosphorylation and dose-dependently decreases Akt phosphorylation, as detected by Western blot analysis (138).

The other anticancer targets of alkaloids are summarized in Table 3.

**Table 3:** Alkaloids and their anticancer mechanisms.

**Mechanism**	**Components**	**Plant origin**	**Cell line**	**References**
HIF-1α protein ↓	Dauricine	*Menispermum dauricum* DC	MCF-7	(139)
VEGF ↓	Dauricine	*Menispermum dauricum* DC	MCF-7	(139)
Ezrin ↓	Berberine	Berberis species	5-8F 6-10B	(140)
MMP-2, 9, 13 ↓	Berberine	Berberis species	A549	(141)
	Piperine	*Piper nigrum*	4T1	(142)
p-Smad2/3 ↓	Berberine	Berberis species	A549	(141)
NF-κB	Noscapine	Opium	KBM-5 HL-60	(141)
	Piperine	*Piper nigrum*	4T1	(142)
	Cryptopleurine	*Boehmeria pannosa*	MDA-MB231 Hep3B	(143)
PI3K/Akt/GSK3β pathway ↓	Tetrandrine	*Stephania* *tetrandra S*. Moore	HT-29	(144)

## Saponins

Saponins are valuable sources with minimal toxic effects and are found in many dietary plants. Saponins are composed of a triterpenoid or steroidal aglycone attached to one or more sugar chains (145). Saponins are divided into two types: triterpenoid saponins and steroidal saponins. Both types have various biological activities, such as anticancer and immunological adjuvant activities (146).

**Figure 6:**
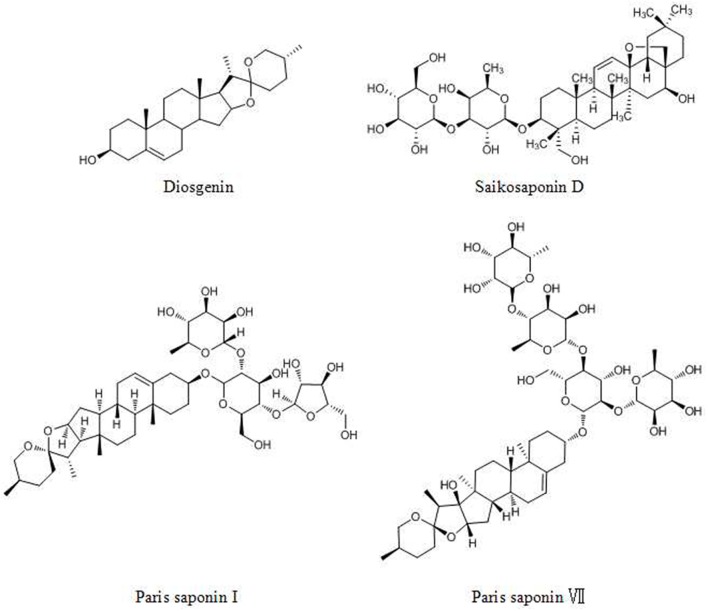
Chemical structures of some saponins.

Diosgenin (DG, Figure 6), a steroidal saponin, has been shown to be an anticancer agent in many tumors. DG acts against cancers via the following pathways and mechanisms: (1) the STAT pathway, (2) activation of caspase-3 and p53, (3) activation of the TRAIL death receptor DR5 and (4) the Wnt-β-catenin pathway (147).

The steroidal saponin of *Paris polyphylla* (Chinese name: Chonglou) has long been used for lung cancer treatment (148). Paris saponin I (PSI, Figure 6) and *Paris polyphylla* steroidal saponins (PPSS) regulate the Bcl-2 family and caspase-3 and -8, inducing apoptosis (149). In addition, PSI and PPSS induce autophagy by the conversion of LC3 I to LC3 II and upregulation of Beclin 1 (150). Paris saponin VII (PS VII, Figure 6), extracted from *Trillium tschonoskii* Maxim, inhibits the migration and invasion of several types of cancer cells via the downregulation of MMP-2 and -9 expression and p38 MAPK phosphorylation in a dose- and time- dependent manner (151).

Saikosaponin D (SSD, Figure 6), prescribed for liver diseases, was reported to exhibit anticancer activities (152, 153). SSD effectively suppresses invasion, metastasis and angiogenesis via the downregulation of TNF-α mediated NF-κB signaling, affecting proteins such as MMP-9, VEGF, c-myc, cyclin D1, ICAM-1, and COX-2. In addition, SSD activates the Ca^2+^/calmodulin-dependent kinase/AMPK/mTOR pathway and attenuates STAT3/HIF-1 pathway signaling, which induces the apoptosis and inhibits the proliferation of cancer cells (154, 155).

**Figure 7:**
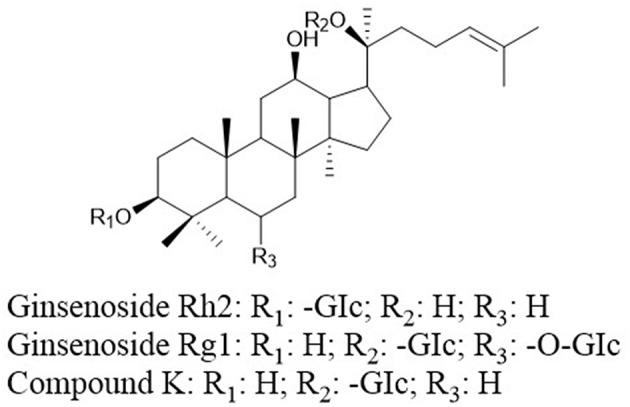
Chemical structures of ginsenosides.

Ginsenosides (ginseng saponins) derived from ginseng were reported to exhibit anticancer effects. Ginsenoside Rh2 (GRh2, Figure 7) and ginsenoside Rg1 (Figure 7) induce apoptosis via activating extrinsic apoptosis pathways by p53-Fas-caspase-8 signaling and the EpoR-mediated JAK2/STAT5 signaling pathway, respectively (156, 157). Moreover, the expression of phosphoglucose isomerase/autocrine motility factor (PGI/AMF) enhances the anticancer effects of GRh2 by attenuating Akt/mTOR signaling (158). A metabolite of ginsenoside compound K (CK, 20-O-D-glucopyranosyl-20(S)-protopanaxadiol, Figure 7) can enhance apoptosis via the ROS-mediated p38 MAPK pathway (159).

## Polysaccharides

Polysaccharides which are abundant in plants, possess anticancer activities, and are being used as immunopotentiators for cancer patients, thus they are relatively ideal anticancer agents (160).

Fucoidans, a class of fucose-enriched sulfated polysaccharides, primarily affect apoptosis-related pathways, as proven both *in vivo* and *in vitro* (161, 162). Apoptotic morphological changes result from the activation of caspases. Caspase-3 and-9 are activated by fucoidan from *Ascophyllum nodosum* (163) mainly composed of 52.1% fucose, 21.3% glucose, 19% sulfate content, and 16.5% xylose. And caspase-7 and -8 are regulated by a sulfated polysaccharide isolated from an enzymatic digestion of *Ecklonia cava* (164). Cell apoptosis induced by S-fucoidan from *Cladosiphon okamuranus* depends on caspase-3 and -7 (165). Other targets involved in apoptotic effects include Bax and Bcl-xL, ERKs, p38, and the PI3K/Akt signaling pathway (166). Fucoidan, from *Cladosiphon novae-caledoniae Kylin*, which is consisted of 73% fucose, 12% xylose and mannose, inhibits invasion and tubule formation via the suppression of MMP-2 and -9 activity and downregulation of VEGF expression in tumor cells (167).

The purified polysaccharide extracted from *Caulerpa lentillifera*, SP1, composed mainly of sulfated xylogalatan and galactose, showed potent immunostimulatory effects by activating macrophage cells through both the NF-κB and p38 MAPK signaling pathways (168). SP1 decreased the levels of IκBα and the NF-κB p65 subunit and increased p38 MAPK phosphorylation, as determined by Western blot assay.

Polysaccharides extracted from *Phellinus linteus* (PL) significantly inhibit cell proliferation by decreasing β-catenin and cyclin D1 expression *in vitro*. In addition, PL inhibits invasion and motility by directly reducing the activity of MMP-2 and -9, with no effect on the gene expression or secretion of MMPs, as indicated by RT-PCR and gelatin zymography (169).

The *Radix astragali* active extract Astragalus polysaccharide (APS) can enhance the immune response by promoting IL-2, IL-6, and TNF-α in H22 tumor-bearing mice. The effects on the immune response are involved in the inhibition of cancer. In addition to the immune response, the anticancer mechanism involves apoptosis, cell cycle arrest, Akt phosphorylation, Bcl-2 and Bax, caspase-3 and -9, p53 and PTEN (163, 170).

The polysaccharides obtained from enzymatic digestion by Celluclast enzyme digest (CCP) suppresses the activation of NF-κB p50 and p65 and the phosphorylation of p38 MAPK in macrophages (171).

*Ganoderma lucidum* (*G. lucidum*) polysaccharides (GLPs) can inhibit growth in many types of cancer by inducing apoptosis through FOXO3a-TNF-α-NF-κB signalway (172).

## Conclusion

**Figure 8:**
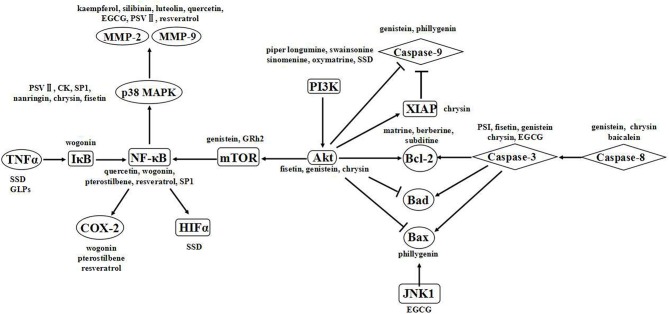
Signaling pathways of the natural products.

Natural compounds offer a great diversity of chemical structures that are likely important in cancer therapeutics (18). Many studies have shown that phytochemicals influence targets and signaling pathways involved in oncogenesis and tumor progression such as proliferation, invasion, metastasis and angiogenesis (173). Different components have various anticancer activities. (1) Alkaloids, with low bioavailability and poor water solubility, have difficulty to reaching the intended target. Moreover, the toxicity of alkaloids cannot be ignored, primarily target apoptosis-related pathways (174). (2) Flavonoids can affect the development of colon, lung, esophageal, stomach and endometrial cancer, with minimal acute toxic effects because of their poor water solubility accompanied by their rapid digestion (17, 175). Polyphenols primarily target pathways related to proliferation, apoptosis, invasion and metastasis. (3) Polysaccharides and saponins effectively modulate the immune response rather than directly inducing cell death. Polysaccharides primarily affect apoptosis-related pathways, while saponins affect apoptosis-related and invasion- and metastasis-related pathways (176). The anticancer effects of these compounds are associated with multiple targets (Figure 8) (176). Signaling pathways are believed to be associated with specific chemical structures, and this association is critical for continuing drug development.

## Author Contributions

LS, WZ, and HZ wrote the article. QG, WY, BL, ZS, and SG provided critical advises of the article. ZS and RC provided final revision.

### Conflict of Interest

The authors declare that the research was conducted in the absence of any commercial or financial relationships that could be construed as a potential conflict of interest.
